# Low-dose decitabine combined with camrelizumab for refractory/relapsed mycosis fungoides/Sézary syndrome: a retrospective exploratory study

**DOI:** 10.3389/fonc.2026.1851475

**Published:** 2026-08-06

**Authors:** Shan Meng, Longfei Zhu, Bin Peng, Chen Tu, Yilin Zhang, Xingmei Cao, Liufang Gu

**Affiliations:** 1 Department of Hematology, the Second Affiliated Hospital of Xi’an Jiaotong University, Xi’an Shaanxi, China; 2 Department of Dermatology, the Second Affiliated Hospital of Xi’an Jiaotong University, Xi’an Shaanxi, China

**Keywords:** camrelizumab, cutaneous T-cell lymphoma, demethylation, immune checkpoint inhibitors, mycosis fungoides/Sézary syndrome

## Abstract

**Background:**

Mycosis fungoides (MF) and Sézary syndrome (SS) are the main subtypes of cutaneous T-cell lymphomas that are characterized by clinical heterogeneity and are difficult to cure. Objective: This retrospective study evaluated the efficacy and immunomodulatory effects of low-dose decitabine combined with camrelizumab, a novel PD-1 monoclonal antibody used to treat patients with refractory advanced MF/SS.

**Methods:**

The enrolled patients received decitabine (10 mg/d, days 1–5) combined with camrelizumab (200 mg, day 7) every 4 weeks. The response was assessed and peripheral blood was analyzed for T-cell subsets, cytokines, Lactate Dehydrogenase (LDH), β2-microglobulin levels, and lymphocyte parameters. Adverse events were monitored.

**Results**

From February 2021 to May 2025, 14 patients were enrolled in the study (3 SS, 1 folliculotropic MF, and 6 with large-cell transformation). The objective response rate was 71.4% (10/14), with 5 complete (CR) and 5 partial responses (PR). At a median of 13.5 months follow-up, 3 patients maintained sustained CR. Common adverse events were grade 1–2 leukopenia (21.4%), elevated transaminases (28.6%), and camrelizumab-related mucocutaneous capillary proliferation (50.0%). Exploratory immunophenotyping showed a progressive decline in the CD4^+^/CD8^+^ ratio, lower baseline LDH, and absolute lymphocyte counts in responders, whereas non-responders had significantly higher IL-5 and IL-13 levels (*P* < 0.05).

**Conclusion:**

The combination regimen showed encouraging preliminary activity and tolerability in this small retrospective cohort, although the efficacy of SS remains suboptimal. These findings are hypothesis-generating and require prospective validation.

## 1 Introduction

Mycosis fungoides (MF) and Sézary syndrome (SS) are the most common type of cutaneous T-cell lymphoma (CTCLs), which typically present as skin-limited patches and plaques in sun-protected areas and have an indolent clinical course, However, advanced-stage MF and SS exhibit aggressive features, with clinical presentations that include lymph node involvement, visceral involvement, the presence of neoplastic cells in the peripheral blood, and marked immune dysregulation. These complex manifestations increase the diagnostic difficulty and, together with the lack of specific and effective treatment options, ultimately lead to reduced overall survival (OS) (1, 2).

Currently, there is no definite and effective treatment for MF/SS in the advanced stage. In addition to phototherapy, retinoic acid, interferon, and other skin treatments, cytotoxic drugs such as methotrexate, liposome doxorubicin, gemcitabine have certain effects on MF (3–5). Systemic and targeted options such as brentuximab vedotin, histone deacetylase inhibitors, mogamulizumab, and allogeneic hematopoietic stem cell transplantation in selected eligible patients are also used for relapsed/refractory or advanced disease (6, 7). Nevertheless, durable complete remissions remain uncommon across available treatment options.

Emerging evidence suggests that the pathogenesis of MF/SS involves T-cell exhaustion and immune evasion by malignant T cells, which is characterized by upregulation of immune checkpoint molecules and a shift toward a Th2-dominant tumor microenvironment. PD-1 blockade has shown activity in relapsed/refractory MF/SS, but PD-1 signaling in CTCL is complex as checkpoint molecules may be expressed by reactive immune cells and malignant T cells (8–10). Because the pathogenesis of advanced-stage MF and SS involves immune evasion and the participation of PD-1/PD-L1 expression, we selected low-dose decitabine combined with camrelizumab for the treatment of advanced-stage MF and SS. We hypothesized that hypomethylating agents may partly reverse exhaustion-associated epigenetic programs and improve immune responsiveness (11–13). Our study aimed to evaluate the therapeutic activity, safety profile, and immunologic effects of decitabine plus camrelizumab in patients with relapsed or refractory MF/SS.

## 2 Materials and methods

### 2.1 Study design

We retrospectively selected patients with stage IIB-IV relapsed/refractory MF/SS who had received at least 2 cycles of decitabine combined with camrelizumab and were eligible for at least one efficacy evaluation between February 2021 and May 2025. This criterion was used because response assessment was scheduled after induction therapy. It must be acknowledged that the exclusion of patients with rapid disease progression or early toxicity could lead to an overestimation of response rates. These patients had all previously received at least one line of therapy and exhibited refractory disease characteristics, defined as persistent, progressive, or relapsed disease requiring subsequent systemic treatment. The study was conducted at The Second Affiliated Hospital of Xi’an Jiaotong University and was approved by the institutional ethics committee (Approval No (2025).261). All patients provided written informed consent before treatment. The study was conducted in accordance with the Declaration of Helsinki and relevant national regulatory requirements.

### 2.2 Patients

Patients with histopathologically confirmed relapsed/refractory MF/SS, clinically staged as IIB-IV, who received at least 2 cycles of the treatment regimen, were selected for analysis.

### 2.3 Treatment

Patients received decitabine at a dose of 10 mg/d via intravenous drip on days 1–5 of each 4-week cycle, followed by camrelizumab 200 mg administered intravenously on day 7 of each cycle.

### 2.4 Efficacy assessment

Treatment efficacy was assessed every 2–3 cycles. Clinical response in MF/SS was evaluated using the global response score, established by the joint consensus recommendations of the European Organization for Research and Treatment of Cancer, the International Society for Cutaneous Lymphomas, and the United States Cutaneous Lymphoma Consortium (1). This composite scoring system incorporates assessments of skin, lymph nodes, peripheral blood, and visceral involvement. Based on the comprehensive assessment of all involved sites, the patient’s overall response category was determined (CR: complete response, PR: partial response, SD: stable disease, PD: progressive disease). Skin involvement was assessed using the modified Severity Weighted Assessment Tool (mSWAT) (1). Lymph node and visceral involvement were evaluated using contrast-enhanced computed tomography (CT) every 2–3 treatment cycles. Blood and bone marrow involvement were assessed in patients with SS through peripheral blood smear and flow cytometry-based immunophenotyping every 2–3 cycles.

### 2.5 Adverse events

Adverse events (AEs) were monitored and graded according to the National Cancer Institute Common Terminology Criteria for Adverse Events, version 5.0.

### 2.6 Mechanistic explorations

To investigate the immunomodulatory effects of treatment, peripheral blood specimens were collected at baseline and after each treatment cycle for flow cytometric analysis and biochemical assessment.

#### 2.6.1 Flow cytometry

Peripheral T-cell subsets, including CD4^+^ T cells, CD8^+^ T cells, regulatory T cells (Tregs), and the CD4/CD8 ratio, were analyzed using fluorescence-activated cell sorting at baseline and during treatment when samples were available. Cells were washed and incubated with staining buffer (BD Biosciences) at 4°C. Lymphocytes were initially gated according to forward- and side-scatter characteristics, followed by T-cell subset analysis. Data acquisition was performed by a fluorescence-activated cell sorting (FACS)-Calibur cytometer (BD Biosciences) and analyzed by FlowJo software.

#### 2.6.2 Lactate dehydrogenase assay

Lactate Dehydrogenase (LDH) activity was measured at baseline and after each treatment cycle using a colorimetry method. EDTA-K2 anticoagulated whole blood was centrifuge at 4 °C and 1500 ×*g* for 10 min to separate plasma. A working reagent containing substrate, NAD^+^, and coupling agent was prepared according to the kit instructions. Plasma samples or standards were incubated with the working reagent at 37 °C for 20–30 minutes in the dark. The reaction was terminated, and absorbance was measured at 490–570 nm using a spectrophotometer. LDH activity was calculated against a standard curve after background subtraction.

### 2.7 Statistical analysis

All statistical analyses were performed using GraphPad Prism software (v.10.1.2; GraphPad Software, San Diego, CA, USA). Due to the small sample size, the analyses were predominantly descriptive and hypothesis-generating. Categorical variables were compared using Fisher’s exact test, as appropriate. Continuous variables were analyzed using the independent-sample t-test or the Mann–Whitney U-test, depending on data distribution. No Cox regression or multivariable survival analysis was performed because of the limited number of patients and events. All tests were two-sided, and *P* < 0.05 was considered statistically significant.

## 3 Results

Fourteen patients with confirmed MF or SS were enrolled between February 2021 and May 2025. The cohort comprised 10 males and 4 females, with a median age of 55.5 years (range, 37–83 years). Among them, 3 patients had SS, 1 patient had folliculotropic MF (fMF), and 6 showed histopathologic evidence of large-cell transformation. Clinical variants included advanced stage MF/SS (n = 14). According to corrected Tumor-Node-Metastasis-Blood (TNMB) staging, 11 patients had stage IIB disease and 1 had stage IVA1 SS and 2 had IVB SS. Baseline characteristics, including TNMB stage, clinical subtype, large-cell transformation status, body surface area (BSA) involvement, and mSWAT scores, are summarized in **Table 1**.

**Table 1:** Baseline characteristics of patients.

Patient	Age (y)	Sex	TNMB stage	MF/SS stage	Clinical stage	Large-cell transformation	BSA involvement (%)	Baseline mSWAT	Prior therapies
Pt 1	59	M	T3N2M0B0	IIB	Tumor	Yes	8	22	4
Pt 2	58	M	T3N2M0B0	IIB	Tumor	Yes	18	72	3
Pt 3	55	M	T3N0M0B0	IIB	Tumor	No	15	40	3
Pt 4	59	F	T4NxM1aB2	IVB(SS)	Tumor	Yes	98	196	3
Pt 5	52	F	T4NxMxB2	IVA1 (SS)	Tumor	Yes	100	218	2
Pt 6	56	M	T3N0M0B0	IIB	Tumor	No	23	92	3
Pt 7	39	M	T3N0M0B0	IIB	Tumor	Yes	33	112	4
Pt 8	47	M	T3N2bM0B0	IIB	Tumor	No	44	96	2
Pt 9	48	M	T3N2bM0B0	IIB	Tumor	No	29	82	3
Pt 10	81	M	T3NxM0B0	IIB	Tumor	No	2	4	2
Pt 11	83	M	T3NxM0B0	IIB	Tumor	No	31	124	1
Pt 12	37	M	T3NxM1aB2	IVB(SS)	Tumor	No	37	148	3
Pt 13	65	F	T3N0M0B0	IIB	Tumor	No	8	32	2
Pt 14	37	F	T3N1aM0B0	IIB	Tumor	Yes	41	126	3

M, male; F, female; TNMB, Tumor-Node-Metastasis-Blood; BSA, Body Surface Area; SWAT, Severity Weighted Assessment Tool.

### 3.1 Efficacy

All 14 patients underwent efficacy evaluation following 2–3 cycles of treatment. The objective response rate (ORR) was 71.4% (10/14), including 5 patients (35.7%) who achieved CR and 5 patients (35.7%) with PR. SD was observed in 2 patients: 1 with SS and 1 with fMF. Furthermore, 1 patient with SS experienced PD, whereas another with SS showed no response after 3 cycles and subsequently died from *Pseudomonas aeruginosa* sepsis.

**Figure 1:**
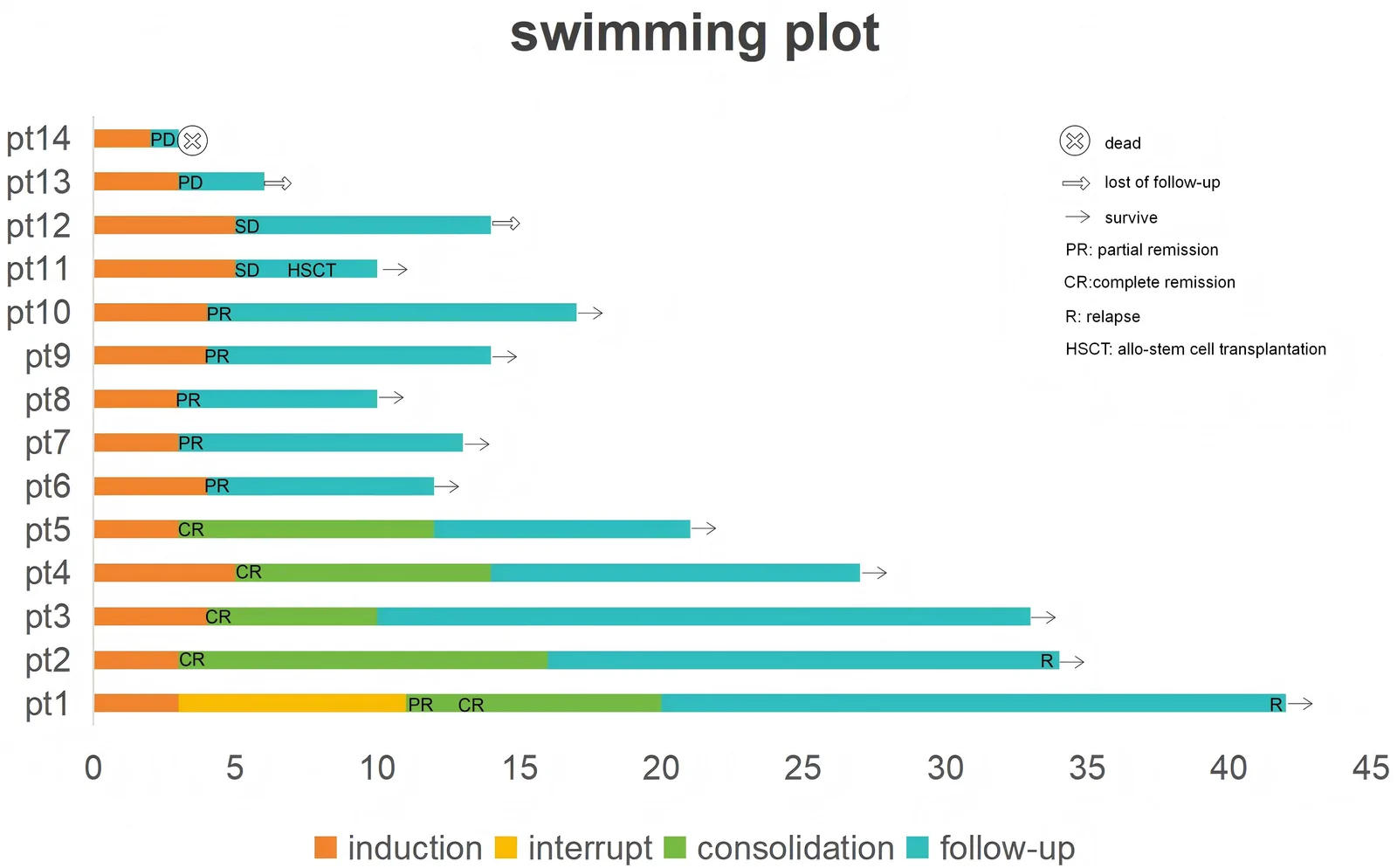
Swimming plot of the 14 patients CR, complete response, PR, partial response, R, relapse, HSCT, allo-stem cell transplantation, SD, stable disease. Alt text: Swimming plot showing treatment phases and clinical responses for the 14 patients. pt1–pt5 are patients with advanced MF/SS over time (0–40 on the x-axis). Each horizontal bar represents a patient, with segments colored to indicate treatment phases: orange for induction, yellow for treatment interruption (interrupt), green for consolidation, and blue for follow-up. Clinical response labels include CR (complete response), PR (partial response), and R (relapse). pt1 has an orange interrupt segment, followed by consolidation and follow-up with relapse. pt2–pt5 show induction, consolidation, and follow-up, with CR noted during consolidation; pt2 and pt1 also show relapse (R) during follow-up. Pt6 to 10 achieved PR after induction therapy and were switched to the next-line treatment; pt11 attained stable disease (SD) following induction therapy and subsequently underwent allogeneic hematopoietic stem cell transplantation (HSCT). Pt12 and 13 were lost to follow-up thereafter, and pt14 developed progressive disease (PD) and died of sepsis.

At a median follow-up of 13.5 months, 3 patients remained in sustained CR. Among the three patients with SS, overall efficacy was limited: 1 patient was lost to follow-up after persistent SD, 1 died of sepsis due to extensive skin infection, and 1 underwent allogeneic blood hematopoietic stem cell transplantation after SD. The swimmer plot illustrating the therapeutic efficacy changes of the 14 patients is shown in **Figure 1**.

**Figure 2:**
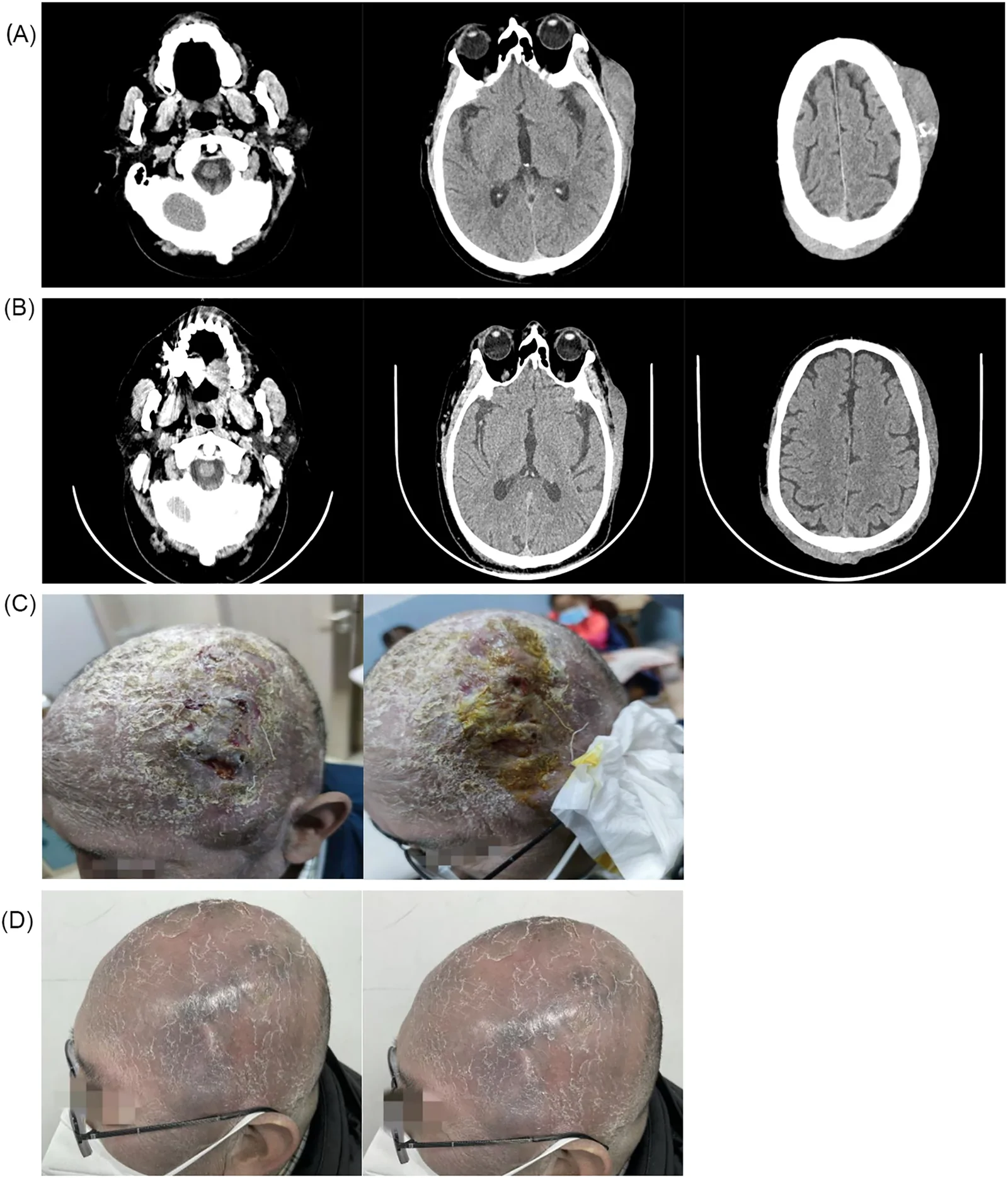
Pre- and post- treatment imaging and physical signs of patient 1. **(A, C)** Baseline cranial and parotid computed tomography (CT) images as well as physical signs; **(B, D)** cranial and parotid CT images obtained after a 2-cycle treatment, during an off-treatment follow-up period of up to 8 months, as well as physical signs. Alt text: Figure showing pre- and post-treatment imaging and clinical findings for a patient with scalp lesions. **(A)** Pre-treatment axial computed tomography(CT) scans of the head at three levels, revealing a well-defined, hyperdense, expansile lesion in the left parietal scalp, with associated calvarial erosion. **(B)** Post-treatment axial CT scans of the head at corresponding levels, demonstrating significant regression of the scalp lesion and resolution of calvarial erosion. **(C)** Pre-treatment clinical photographs of the patient’s scalp, showing a large, ulcerated, crusted lesion with surrounding erythema and scale. **(D)** Post-treatment clinical photographs of the patient’s scalp, showing complete healing of the lesion, with only residual scarring and mild desquamation remaining.

**Figure 3:**
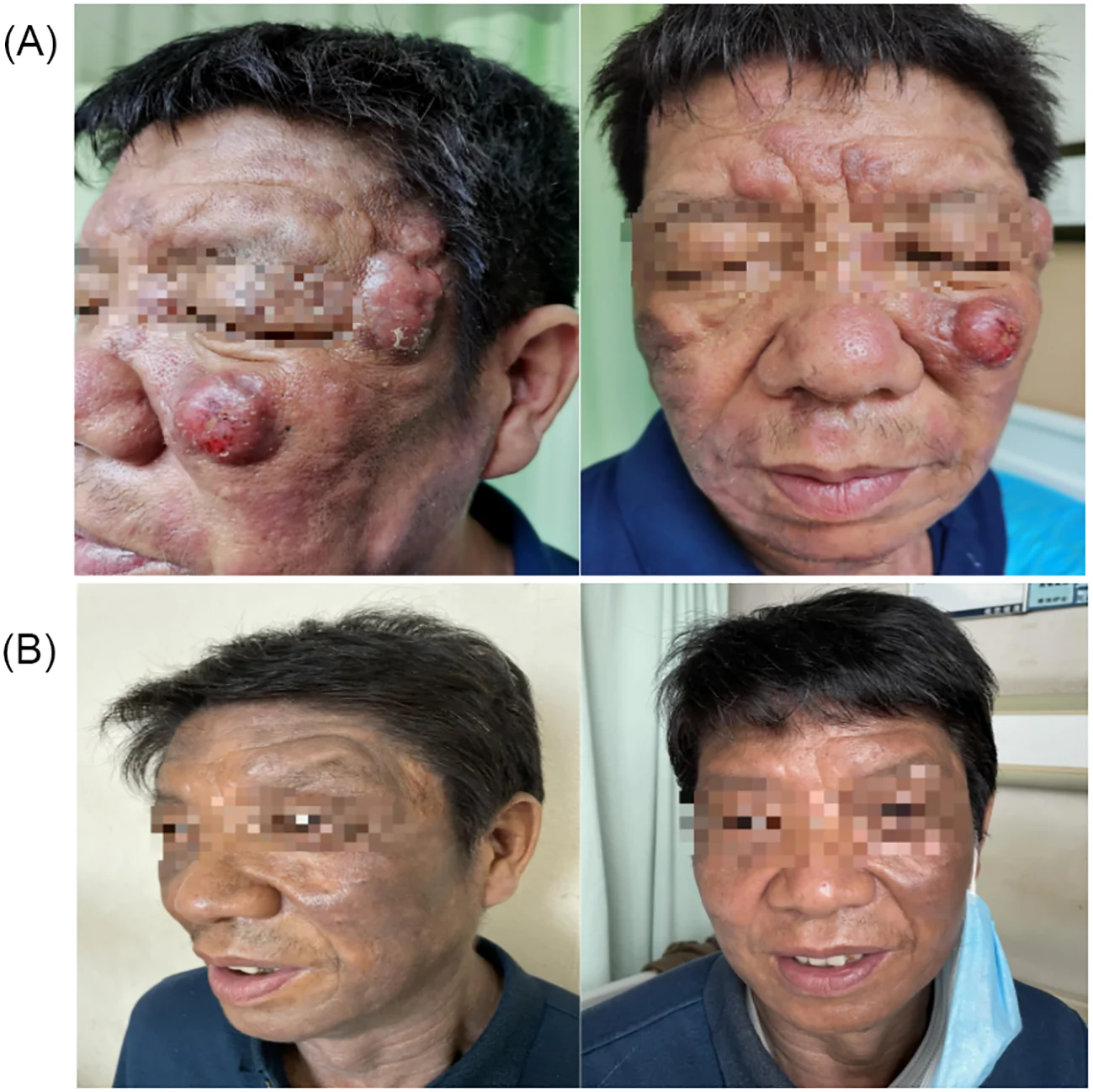
Pre- and post-2 cycles of treatment physical signs of patient 2. Alt text: Clinical photographs showing the patient’s facial lesions before and after treatment. **(A)** Pre-treatment photographs displaying multiple erythematous, nodular, and ulcerated lesions on the forehead, cheeks, and nose, with significant facial swelling and discoloration. **(B)** Post-treatment photographs demonstrating complete resolution of the facial nodules and ulcers, with only residual hyperpigmentation and mild skin texture changes remaining.

Notably, 2 cases demonstrated marked clinical responses to the treatment regimen and are illustrated in **Figure 2** and **3**. Patient 1 presented with extensive ulcerated masses involving the scalp and parotid region before enrollment. Following one cycle of decitabine combined with camrelizumab, the ulceration had nearly completely resolved, and the tumor mass significantly regressed. However, treatment was subsequently interrupted for approximately 8 months due to the COVID-19 pandemic. Upon re-evaluation, the patient maintained a PR. CR was achieved after two additional cycles of regular treatment, which has been maintained to date. Patient 2, who had experienced disease progression after multiple prior lines of therapy, achieved CR after two cycles of treatment. This remission has also been maintained through subsequent follow-up.

### 3.2 Adverse effects

A total of 56 treatment cycles were administered across all patients. Treatment was generally well tolerated and most of AEs resolved spontaneously with routine monitoring, as shown in **Table 2**.

**Table 2:** Adverse events in the enrolled 14 patients.

Adverse Event	N = 14 (%), Grade 1–2	N, Grade ≥3
Hematological AE		0
Leukopenia:	3 (21.4%)	
Thrombocytopenia	1 (7.1%)	
Non-hematological AE		
Elevated aminotransferases	4 (28.6%)	
Gastrointestinal discomfort	2 (14.3%)	
Mucocutaneous capillary proliferation	7 (50.0%)	
Hypothyroidism	2 (14.3%)	
Bulbar conjunctival hyperplasia	1 (7.1%)	
Infusion-related reactions	4 (28.6%)	

AE, adverse event.

### 3.3 Mechanism exploration

#### 3.3.1 Peripheral T cell subset monitoring

Peripheral T-cell subset analysis was performed in 12 patients over the first five treatment cycles, with all values presented as medians. Among them, 8 patients achieved ORR, and 4 were classified as non-responders. In the ORR group, a progressive decrease in CD4^+^ T-cell counts and the CD4^+^/CD8^+^ ratio was observed, although CD8^+^ T-cell counts did not consistently increase. In contrast, no consistent trend was observed in the non-responder group (*P* < 0.05; **Supplementary Figure 1**). Among the three patients with Sézary syndrome, two showed persistently elevated CD4^+^ T-cell counts, low CD8^+^ T-cell levels, and sustained increases in the CD4^+^/CD8^+^ ratio, consistent with disease progression. These exploratory findings suggest that that peripheral T-cell subset dynamics, particularly changes in the CD4^+^/CD8^+^ ratio, may warrant further evaluation as candidate response-associated markers.

#### 3.3.2 Th1/Th2 cytokine monitoring

Cytokine profiling was performed in six patients from baseline to the third treatment cycle, including assessment of Th1 (IFN-α) and Th2 cytokines (IL-4, IL-10, IL-5, IL-13). Among these, three patients achieved ORR and three were non-responders. No statistically significant differences were observed between groups in IL-4, IL-10, or IFN-α level. However, IL-5 levels were significantly higher in the non-responder group compared with the ORR group (*P* < 0.05; **Supplementary Figure 2A**). Similarly, IL-13 levels were significantly increased in non-responders (*P* < 0.05; **Supplementary Figure 2B**). Given the very small number of evaluable patients, these findings should be interpreted as preliminary and hypothesis-generating and may reflect the known Th2-skewed profile of advanced CTCL.

#### 3.3.3 Other biochemical indicators

**Figure 4:**
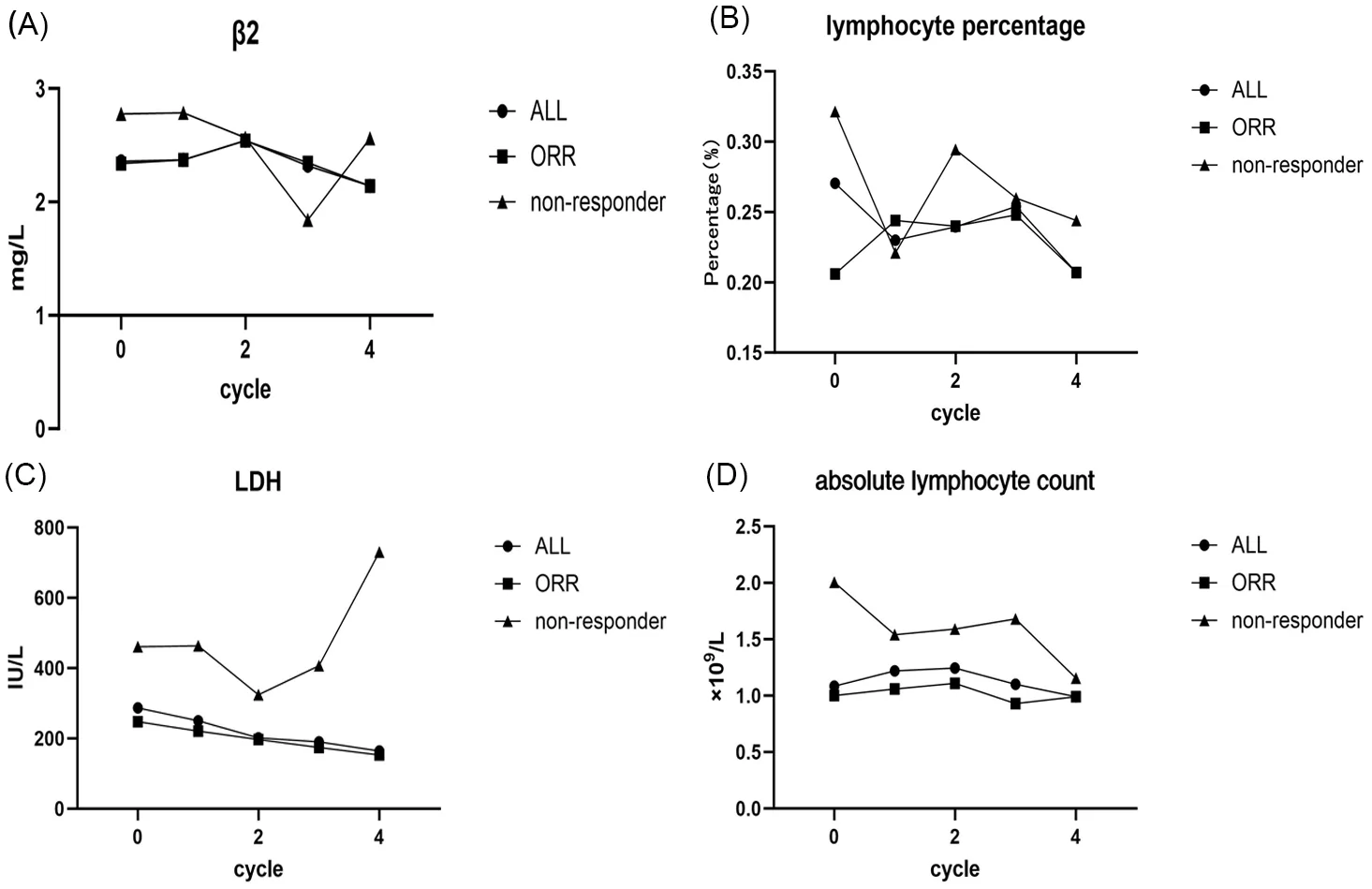
Changes in biochemical indicators during the treatment. **(A)** Changes in β2-microglobulin levels; **(B)** Percentage changes in lymphocyte levels; **(C)** Trend in changes in LDH levels; and **(D)** trend in changes in absolute lymphocyte count. Alt text: Line graphs showing the dynamic changes of β2-microglobulin, lymphocyte percentages, LDH, and absolute lymphocyte count over treatment cycles (0 to 4) in three patient groups: ALL (circles), ORR (squares), and non-responders (triangles). Panel **(A)** displays β2-microglobulin levels (mg/L), with all groups showing fluctuations around 2–3 mg/L. Panel **(B)** shows lymphocyte percentage (%), with the non-responder group exhibiting a peak at cycle 2. Panel **(C)** presents LDH levels (IU/L), in which the non-responder group shows a sharp increase at cycle 4. Panel **(D)** illustrates absolute lymphocyte count (×10^9^/L), with the non-responder group maintaining the highest counts throughout the treatment period.

Biochemical data from baseline to the fourth treatment cycle were collected from 13 subjects, including LDH, β2-microglobulin, lymphocyte percentage, and absolute lymphocyte count. Among them, ten patients were classified as responders and three as non-responders. At baseline, the β2-microglobulin levels and lymphocyte percentage in the ORR group were noticeably lower than those in the non-responders, though these differences did not reach statistical significance. A declining trend in β2-microglobulin levels was observed during treatment in the ORR group (**Figures 4A, B**).

Baseline LDH levels were significantly lower in responders compared with non-responders (*P* < 0.05; **Figure 4C**). Similarly, absolute lymphocyte count at baseline was also significantly lower in the ORR group (*P* < 0.05; **Figure 4D**). These exploratory findings suggest that lower baseline levels of LDH and absolute lymphocyte count may be associated with better treatment response, However, these results derive from a small cohort and should not be considered confirmatory.

## 4 Discussion

Currently, numerous targeted therapeutic agents are available for the treatment of MF/SS. Romidepsin, a histone deacetylase inhibitor, was FDA-approved for relapsed/refractory CTCL based on a Phase II trial showing an ORR of 34% (14). However, in real-world studies of romidepsin, the ORR was 25%, and none of the treated patients achieved a CR. Mogamulizumab demonstrated superior median progression-free survival of 7.7 months versus 3.1 months with vorinostat (15). Brentuximab vedotin, approved for CD30-positive MF/SS after at least one prior line of therapy, achieved an ORR of 56.3% and CR rate of 16% in a Phase III trial with a median follow-up of 22.9 months (16). These studies provide clinical context, but they should not be directly compared with the present small retrospective single-arm series.

Several studies have demonstrated that the microenvironment plays a critical role in the initiation and progression of MF/SS. In early-stage MF, the infiltrating immune cell population primarily consists of non-malignant cells, including Th1 helper T cells and CD8^+^ cytotoxic T lymphocytes, which may suppress the proliferation of malignant T cells through cytokine secretion (17). The disease course at this stage is often indolent and can persist for decades. As the disease progresses, malignant T cells accumulate in the skin and the immunophenotype of the tumor microenvironment shifts from a Th1- to a Th2-dominant profile, which characterized by increased IL-4, IL-5, IL-10, and IL-13 production, which contributes to an immunosuppressive tumor milieu and increased susceptibility to infections (18). Therefore, it is speculated that the inactivation of T-cell function and the decrease in the number of tumor-infiltrating T lymphocytes may be important mechanisms for the transformation from plaque stage to tumor stage. Additional preclinical evidence suggests that aberrant DNA methylation promotes T-cell exhaustion and impairs the efficacy of PD-1 blockade, whereas hypomethylating agents may reverse this exhaustion and rejuvenate T-cell function (11–13). However, the role of PD-1 signaling in CTCL is complex, because the PD-1 pathway molecules may be expressed by malignant T cells as well as reactive immune cells, and the biologic effect of checkpoint blockade may vary by disease context (9, 10).

Based on the above theoretical rationale, low-dose decitabine was combined with camrelizumab to explore whether this immuno-epigenetic strategy could produce meaningful clinical efficacy in patients with MF/SS. Our study retrospectively analyzed 14 patients with stage IIB-IV refractory MF/SS who had received at least two cycles of low-dose decitabine combined with camrelizumab. The ORR was 71.4%, including a CR rate of 35.7% and PR rate 35.7%. Durable remissions lasting up to 16 months were observed in some patients. The regimen was generally well tolerated, with AEs mainly of grade 1–2 severity. Reactive cutaneous capillary endothelial proliferation is a distinctive side effect of camrelizumab relative to other PD-1 inhibitors, but it is generally of grade 1–2 in severity and is considered safe and manageable.

This combination regimen has shown encouraging preliminary activity with a favorable safety profile, nevertheless, prospective randomized controlled clinical trials are needed for further validation. In a Phase I clinical study evaluating PD-L1 monoclonal antibody combined with the immunomodulatory agent lenalidomide, among the 12 evaluable patients, 7 achieved PR, but no CR was observed, yielding an ORR of 58%. With regard to AEs, 6/12 patients (2 in dose level (DL) 1 [fatigue, radiation recall]; 1 in DL2 [rash]; 3 in DL3 [decreased white blood cell count, pneumonia, and productive cough]) experienced grade 3 AEs, at least possibly attributed to durvalumab and/or lenalidomide (19).

Recently, similar studies have employed PD-1 inhibitors in combination with demethylating agents or/and pralatrexate for the treatment of peripheral and CTCLs. In this study, the 21 evaluable patients achieved ORR 19% (4/21), CR 10% (2/21), PR 10% (2/21), and the disease control rate (defined as the proportion of patients with stable disease or better) was 38% (8/21). However, among the 26 enrolled patients, the pathological subtypes were heterogeneous with only 6 cases of MF. The observed dose-limiting toxicities (DLT) included one DLT each in arms A and B for prolonged grade 3 thrombocytopenia and febrile neutropenia, respectively. Three DLTs were observed in arm C including one patient with grade 3 hyponatremia and rash; one patient with grade 4 thrombocytopenia, neutropenia, and anemia; and one patient with grade 4 neutropenia. Both hematological and non-hematological AEs warrant greater attention (20).

The clinical activity observed in our study is encouraging. However, due to variations in study design, disease subtype, previous therapies, response evaluation criteria, and length of follow-up, caution is required when interpreting cross-trial comparisons.

Immunological profiling in this study suggested that responders exhibited a sustained reduction in the CD4^+^/CD8^+^ ratio, whereas non-responders displayed persistently elevated Th2 cytokines, particularly of IL-5 and IL-13. In patients who achieved CR, an increase in the number of CD8^+^ T cells and a decrease in the CD4^+^/CD8^+^ ratio was observed. In contrast, among the three patients with SS, CD4^+^ T-cell levels remained elevated, and CD8^+^ T-cell expansion was minimal. These observations align with the evolving understanding of the role of the tumor microenvironment in MF. In early disease, Th1 and cytotoxic T-cell dominance exert antitumor effects, whereas advanced MF is characterized by a Th2-skewed immune milieu with impaired cytotoxicity and heightened susceptibility to infection (7, 8, 21, 22). Admittedly, given the exploratory nature of these immune findings, no functional immune assays or tumor tissue analyses were undertaken to confirm the recovery of T-cell function, Th1 polarization, or any causal relationship.

Nevertheless, several limitations of this study should be acknowledged. First, the retrospective single-arm design precluded the inclusion of a control group for comparative analysis; therefore, the relative contribution of decitabine, camrelizumab, or their combination cannot be determined. Second, the limited and heterogeneous sample size, including tumor-stage MF, folliculotropic MF, SS, and large-cell transformation, may have introduced potential bias. Third, the prerequisite of completing at least two cycles and undergoing one efficacy evaluation may have selectively excluded patients with early progression or treatment-limiting toxicity, thus potentially inflating the response estimates. Additionally, the immunological and serum cytokine assessments were confined to descriptive observations, and no functional immune assays or tumor tissue analyses were performed to investigate the immune microenvironment. Despite these limitations, the present study yields encouraging preliminary findings. Future efforts will involve conducting a randomized controlled trial, complemented by exploratory investigations at the cellular and genetic levels, to further elucidate the mechanistic basis of this regimen and to provide a more robust rationale for patient selection and treatment decision-making in clinical practice.

## Data Availability

The raw data supporting the conclusions of this article will be made available by the authors, without undue reservation.
