# Supplementary material for: Low-dose decitabine combined with camrelizumab for refractory/relapsed mycosis fungoides/Sézary syndrome: a retrospective exploratory study
Source: Front Oncol. 2026 Aug 6;16:1851475. doi: 10.3389/fonc.2026.1851475 (PMC13489844; doi:10.3389/fonc.2026.1851475)
Supplement: Supplementary file 1 [file Table1.docx]

Supplementary Material

## Supplementary Figures

**Supplemental Figure 1.** CD4+/CD8 ratio

**Supplemental Figure 2.** Cytokine profiling changes during the treatment A showed trend of IL-5 Changes; B showed trend of IL-13 Changes

**Supplemental Figure legends:**

Supplemental Figure 1. CD4+/CD8 ratio

Alt text: Line graph showing the changes in CD4+/CD8+ ratio over treatment cycles (0 to 5) for three patient groups: ALL (circles), ORR (squares), and non-responders (triangles). The x-axis represents treatment cycles, and the y-axis represents the CD4+/CD8+ ratio. The non-responder group shows a marked peak at cycle 2, while the ALL and ORR groups maintain relatively stable ratios between 1.5 and 2.5 throughout the treatment period.

Supplemental Figure 2. Cytokine profiling changes during the treatment. (**A**) showed trend of IL-5 Changes; B showed trend of IL-13 Changes

Alt text: Line graphs showing the dynamic changes of interleukin levels over treatment cycles (0 to 3) in three patient groups: ALL (circles), ORR (squares), and non-responders (triangles). Panel (**A**) displays IL-5 concentrations (pg/mL), with the non-responder group showing the highest levels and a peak at cycle 2. Panel (**B**) displays IL-13 concentrations (pg/mL), where the non-responder group also exhibits the highest levels, peaking at cycle 1, while the ALL and ORR groups show more modest fluctuations throughout the treatment period.

.
